# Comparison of the Utility of Amplitude–Spectral and Coherence Features of Psychotropic Drugs’ Action on ECoG Signal for Pharmaco-EEG Based Drug Screening in Rats

**DOI:** 10.3390/mps9050123

**Published:** 2026-08-23

**Authors:** Yuriy I. Sysoev, Nikita S. Kurmazov, Darya D. Shitc, Sergey V. Okovityi

**Affiliations:** 1 Laboratory of Motor and Visceral Functions Neuromodulation, Pavlov Institute of Physiology, Russian Academy of Sciences (RAS), 199034 Saint-Petersburg, Russia; 2 Department of Pharmacology and Clinical Pharmacology, Saint-Petersburg State Chemical Pharmaceutical University, 197022 Saint Petersburg, Russia; kurmazov.nikita@pharminnotech.com (N.S.K.); sergey.okovity@pharminnotech.com (S.V.O.); 3 N.P. Bechtereva Institute of the Human Brain, 197022 Saint Petersburg, Russia; 4 Institute of Translational Biomedicine, Saint Petersburg State University, 199034 Saint Petersburg, Russia; darya.shic@spcpu.ru

**Keywords:** pharmaco-EEG, ECoG, rats, principal component analysis, naive Bayesian classifier, amplitude–spectral analysis, coherence analysis

## Abstract

A naive Bayesian classifier (NBC) combined with principal component analysis (PCA) effectively differentiates the dose-dependent effects of certain groups of psychoactive drugs based on their impact on the amplitude–spectral characteristics of electrocorticograms (ECoG) in rats. This approach has been shown to be useful for pharmacological screening of agents with unknown or poorly understood activity. Despite previously obtained optimistic results, classification determination for some drugs was inaccurate, necessitating the search for possible ways to improve the predictive effectiveness of the proposed algorithm. One possible approach would be to use as input quantitative data not only the impact of the psychoactive drugs studied on the amplitude–spectral characteristics of ECoG but also connectivity changes, including the average coherence power of different pairs of leads. **The aim** of this study was to compare the accuracy of NBC in classifying the pharmacological mechanism of action of agents with well-known mechanisms (test set) using pharmaco-EEG data on changes in the amplitude–spectral characteristics of ECoG, coherence, and the combined use of two data sets. **Materials and methods.** Experiments were performed on Wistar rats with chronically implanted ECoG electrodes. The training set, relative to which the effects of the pharmacological agents from the test set were classified, were the matrices of effects of 12 pharmacological agents: the NMDA antagonist dizocilpine, the D2/D3 antagonists haloperidol and sulpiride, the M-anticholinergic tropicamide, the H1/5HT2A receptor blocker hydroxyzine, the acetylcholinesterase inhibitor galantamine, the alpha-2 adrenergic agonist dexmedetomidine, the alpha-2 adrenergic antagonist atipamezole, the adenosine receptor blocker caffeine and the GABA-mimetics aminophenylbutyric acid (phenibut), bromdihydrochlorophenylbenzodiazepine (phenazepam) and 5-ethyl-5-phenyl-2,4,6(1H,3H,5H)-pyrimidinetrione. The test set included various drugs with tropism for the targets of the training set drugs: dopamine receptor antagonists chlorpromazine, droperidol, tiapride and raclopride, H1-histamine blockers diphenhydramine and promethazine, 5-HT2-receptor blockers ritanserin and glemenserin, acetylcholinesterase inhibitor ipidacrine, alpha2-adrenergic receptor antagonist yohimbine, alpha2-adrenergic agonists medetomidine and xylazine, GABA-mimetics 5-ethyl-5-(1-methylbutyl)-2,4,6(1H,3H,5H)-pyrimidinetrione and chloral hydrate. The analysis of the ECoG signal included the calculation of 132 amplitude–spectral characteristics and 75 coherence indicators, which, using the PCA, led to new integrative indicators used for further classification of the NBC. **Results and discussion.** For each drug in the test set, the median similarity probability with a particular group from the training set was calculated, which was used to assess the classification quality. It was found that, when using the amplitude–spectral characteristics of ECoG, the proposed methodological approach allows for the identification of the ECoG effects of several groups of psychoactive drugs, including D2/D3-dopamine, M-cholinergic, H1-histamine, and 5-HT2-serotonin receptor blockers, AChE inhibitors, GABA-mimetics, and alpha-2-adrenergic receptor agonists and antagonists. This approach enabled the correct classification of 18 of 24 groups in the test set. When using changes in coherence indices as the initial data, the classification accuracy also amounted to 18 of 24 groups. When combining the two data sets, the number of correctly identified NBC groups was 20 of 24 groups. When comparing the classification during training (confusion matrix), it was found that coherence data or adding coherence data to the data based on changes in amplitude–spectral characteristics leads to a statistically significant (*p* < 0.01 in both cases) increase in accuracy. **Conclusions.** The obtained data demonstrated high accuracy in classifying the pharmacological activity of the test sample drugs using any of the three compared approaches. Despite the lack of statistically significant differences between them, classification based on the combined dataset demonstrated a higher number of “correct” similarities. This allows us to recommend the approach based on combined data of drug effects on amplitude–spectral characteristics and coherence as the most promising for further studies using pharmaco-EEG screening.

## 1. Introduction

Despite the high prevalence of neurological and mental disorders, many large pharmaceutical companies have paused further investment in psychiatric drug development, citing low likelihood of success and disproportionately high costs. This reflects that it takes nearly nine years to bring a psychotropic drug to market and that the approval rate for psychiatric drugs (success at all development stages leading to regulatory approval) is only 6.2% [1]. High preclinical costs are mainly due to the large number of behavioral tests required for pharmacological screening and extensive use of laboratory animals. Currently, efforts to reduce these costs include using alternative model organisms, such as zebrafish [2,3]. However, even when screening uses simpler organisms, researchers still rely on the classic battery of rodent behavioral tests [4]. In practice, different drug classes often produce superficially similar behavioral phenotypes in the same test. For example, a drug that reduces locomotor activity could be a sedative, anxiolytic, antipsychotic, hypnotic, or simply neurotoxic. Therefore, developing new pharmacological screening methods is a priority in biomedicine.

Pharmacoelectroencephalography (pharmaco-EEG) is an interdisciplinary field combining pharmacology and electrophysiology that studies drug effects on the central nervous system by analyzing changes in brain bioelectrical activity (typically cortical) following administration of psychoactive substances. Interest in pharmaco-EEG has waxed and waned over the past 70 years; early attempts to classify new drugs relied on visually identifying similarities between their EEG effects and those of known compounds [5,6]. This approach depended heavily on researcher experience and attention and was therefore criticized for subjectivity. With the advent of methods for dimensionality reduction, classification, and prediction, and with the availability of selective ligands for neurotransmitter receptors, interest in pharmaco-EEG is growing again [7]. Given a library of EEG effects of known drugs, machine learning algorithms can identify a drug’s class [8,9,10] and potentially suggest its molecular targets [11].

We proposed an approach combining principal component analysis (PCA) and a naive Bayes classifier (NBC) to classify drugs’ pharmacological activity from their effects on ECoG amplitude–spectral characteristics in rats. PCA is a dimensionality-reduction method that uses an orthogonal linear transformation to map data from the original feature space to a lower-dimensional space. The first axis (principal component) is constructed to maximize the variance of the projected data. The second axis is orthogonal to the first and maximizes the remaining variance; subsequent axes follow similarly. These axes are called principal components (PC1, PC2, etc.) [12]. NBC is a simple probabilistic classifier that assumes feature independence, treating each parameter as independent of the others. It is widely used in biomedical research—for example, to detect behavioral events in rodents [13], to diagnose cerebral microbleeds on MRI [14], and to predict drug ototoxicity [15]. We previously demonstrated the effectiveness of combining PCA and NBC for classifying antipsychotic drug effects on EEG parameters in rats [16], for assessing dose–response relationships of antipsychotic effects [17], and for pharmacological screening of drugs with poorly characterized activity [11,18].

Although the proposed approach can identify effects of D2/D3 dopamine antagonists, M-cholinergic agents, H1 histamine blockers, acetylcholinesterase inhibitors, GABAergic agents, and α2-adrenergic agonists from ECoG amplitude–spectral changes in rats, precise classification features remain unestablished for several pharmacological agents [11,16,17,18]. Therefore, further development requires identifying ways to improve the algorithm’s performance. One solution is to use initial data that include both quantitative amplitude–spectral ECoG changes and connectivity measures, specifically average coherence between electrode pairs. Many animal studies report specific coherence changes after administration of psychedelics acting primarily at 5-HT2A receptors [19,20], NMDA receptor antagonists [21], and analgesic/antiepileptic drugs such as pregabalin and mexiletine in models of inflammatory pain and neuropathy [22]. Clinical studies also support drug-specific coherence effects: a systematic review by De Pieri et al. [23] found that reductions in theta, alpha, and beta coherence during schizophrenia pharmacotherapy were associated with positive treatment outcomes. Thus, it can be hypothesized that adding coherence data to the Pharmaco-EEG library we are developing may allow NBC to better distinguish the effects of different groups of psychotropic drugs from each other.

Thus, **the aim of this study** was to compare NBC accuracy in classifying the pharmacological mechanism of action of agents with well-known mechanisms (test set) using pharmaco-EEG data from ECoG amplitude–spectral changes, coherence changes, and the combined dataset. If high classification accuracy is achieved, it can be assumed that the proposed method will perform equally well on agents with an unknown mechanism of action.

## 2. Materials and Methods

Animal experiments were performed in accordance with Directive 2010/63/EU of the European Parliament and Council (22 September 2010), the principles of the Basel Declaration, and Recommendation No. 33 of the Board of the Eurasian Economic Commission (14 November 2023) “On the Guidelines for Working with Laboratory (Experimental) Animals in Preclinical (Non-Clinical) Studies”. The experimental protocol was approved by the Bioethics Committee of Saint Petersburg State Chemical and Pharmaceutical University of the Ministry of Healthcare of the Russian Federation (application protocol R-PEEG2-SA-2022, 15 February 2022). All measures were taken to reduce animal numbers and to minimize suffering.

### 2.1. Animals

The study used 157 male Wistar rats, aged 3 months and weighing 250–300 g. All animals were obtained from the Rappolovo Breeding Center (Kurchatov Institute, Leningrad region, Russia). Rats were housed five per cage at 20–22 °C with a 12 h light/12 h dark cycle. Animals had ad libitum access to food and water throughout the study. Rats were quarantined for 14 days before experiments.

### 2.2. Manufacturing and Implantation of ECoG Electrodes

The manufacturing steps for nichrome ECoG electrodes, the implantation protocol, and postoperative care procedures were described previously [24]. Anesthesia was induced with tiletamine–zolazepam (30 mg/kg, intramuscular; Zoletil 100, Virbac, Carros, France). Upon reaching the stage of deep anesthesia, the rats were shaved and treated with iodine solution on the head surface, after which they were fixed in a stereotactic apparatus (RWD Life Science Inc., Shenzhen, China). Before further manipulations, the eyes were covered with carbomer 974P (Oftagel^®^, Santen Oy, Tampere, Finland) to prevent corneal drying. The incision of the scalp was made in the rostrocaudal direction from the point between the eyes to the beginning of the cervical region. After preparation of the skull surface (removal of the muscular-fascial layer, periosteum, coagulation of bleeding areas), holes of appropriate diameters were drilled for electrodes and fixation screws (drill immersion depth—up to 1 mm). To prevent heating of the brain, drilling was performed at short intervals. Then, the fixation screws and corticographic electrodes were implanted. The recording electrodes were evenly and symmetrically distributed on the surface of the cerebral hemispheres. Next, the electrodes were covered with dental plastic using a syringe and spatula. The skin incision was then sutured, and the sutures and adjacent area were antiseptically treated. Electrodes FP1 and FP2 were placed over the primary motor cortex (AP = 0.0 mm; ML = ±2.5 mm; DV = 1.0 mm), C3 and C4 over the primary somatosensory cortex above the hippocampus (AP = −4.0 mm; ML = ±2.5 mm; DV = 1.0 mm), and O1 and O2 over the secondary visual cortex (AP = −7.0 mm; ML = ±2.5 mm; DV = 1.0 mm). The reference electrode was implanted in the nasal bone, and the ground electrode was placed subcutaneously in the neck.

### 2.3. Postoperative Care Procedures

Operated rats were kept in individual cages (type III for BIO. A.S.^®^ IVC systems, Bioscape/Zoonlab GmbH, Castrop-Rauxel, Germany) with free access to water and food during the entire study period. Animal condition was assessed immediately after recovery from anesthesia and then twice daily (morning and evening); sutures were treated with 5% iodine in alcohol when necessary. To prevent infection and relieve postoperative pain, animals received Bicillin-3 (5000 U/kg, subcutaneously; OAO Sintez, Kurgan, Russia) and ketoprofen (2.5 mg/kg, subcutaneously once daily for 3 days; OOO Velpharm, Kurgan, Russia). To prevent dehydration, rats received 0.9% sodium chloride subcutaneously (5 mL once daily; OOO Grotex, Saint Petersburg, Russia) for the first 3 days after surgery.

### 2.4. Registration of ECoG

**Figure 1:**
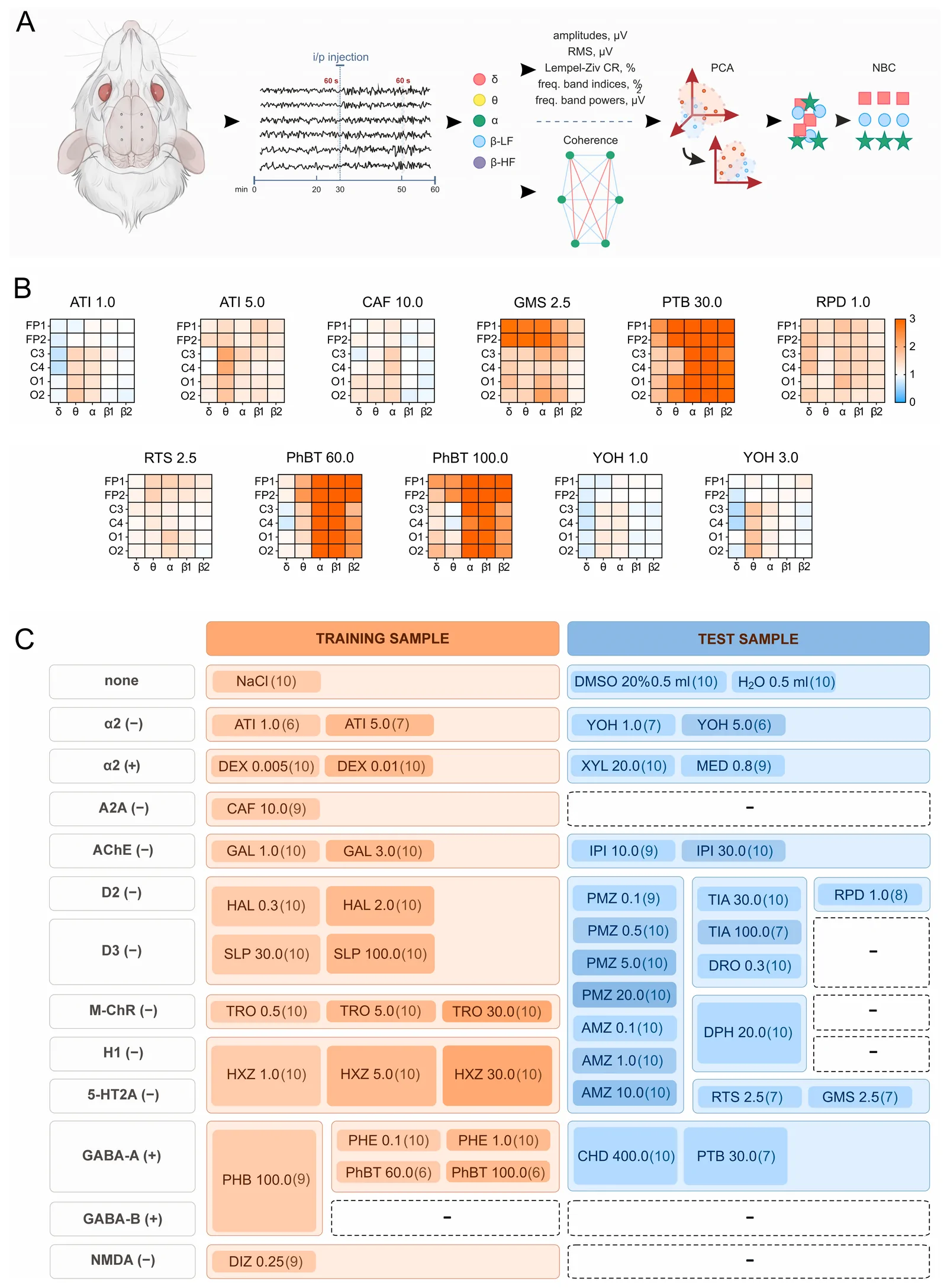
(**A**)—Experimental stages; i/p—intraperitoneal; RMS—root mean square deviation; CR—compression ratio; LF—low frequency; HF—high frequency; PCA—principal component analysis; NBC—naive Bayes classifier; Ref—reference electrode. The datasets for NBC were the ratios of amplitude–spectral parameters ~20th min after administration to corresponding values before administration (~30th min of baseline recording) and difference between coherence values ~20th min after administration to corresponding values before administration (~30th min of baseline recording). For each group, 6–10 recordings were made. (**B**)—Heat maps of median changes in mean power for δ, θ, α, and β frequency bands in leads FP1, FP2, C3, C4, O1, and O2 after administration of novel agents which were added to the previously collected [11] training and test samples: atipamezole (ATI), caffeine (CAF, glemanserin (GMS), 5-ethyl-5-(1-methylbutyl)-2,4,6-(1H,3H,5H)-pyrimidinetrione (PTB), raclopride (RPD), ritanserin (RTS), 5-ethyl-5-phenyl-2,4,6(1H,3H,5H)-pyrimidinetrione (PhBT) and yohimbine (YOH). (**C**)—Agents and their doses used as a training and test set. (+)—agonist/positive allosteric modulator; (-)—antagonist/inhibitor. Note that although SLP and HAL, as well as PhBT and PHE, fall into common groups (D2/D3 blockers and GABA-A agonists, respectively), these drug pairs have profound differences in their mechanisms of action.

ECoG recordings were performed at least one week after implantation using an 8-channel encephalograph (Neuron-Spectr-1, OOO Neurosoft, Ivanovo, Russia) with a bandwidth of 0.5–35 Hz and a sampling frequency of 500 Hz. ECoG was recorded simultaneously with video monitoring of behavior in the home cage under artificial lighting. Recording duration was 1 h, comprising 30 min of baseline activity (before drug or saline administration) and 30 min post-injection (Figure 1A). Two 60 s segments were selected for analysis: one immediately before administration and one 20 min after. Based on previous studies [16,17] and our behavioral-testing experience, most centrally acting psychoactive drugs administered intraperitoneally exhibit pronounced effects within 20 min. Selected segments containing ECoG recorded without motor activity (locomotion, rearing, grooming, scratching) can confound detection and differentiation of drug effects on brain bioelectrical activity [25]. It is precisely because of the high frequency of the above-mentioned behavioral acts in some rats that we selected 60 s fragments of recordings for analysis, i.e., collecting fragments of longer duration from such animals is challenging. The classification and prediction approach described here and previously [16,17] does not constrain the duration of selected ECoG segments. However, because some rats exhibited high background activity requiring removal of movement artifacts, a 60 s segment was chosen as optimal.

### 2.5. Administered Drugs

All drugs for ECoG recording were administered intraperitoneally after dissolution in water for injection to the required concentration, when needed. The selection of specific drugs for the training set was driven by the desire to record the effects of drugs acting on the most common pharmacological targets of psychotropic medications (e.g., dopamine D2 receptors, M-cholinergic receptors, and others). Furthermore, the drugs in the training set should have the most selective pharmacological action; for example, this is why haloperidol, rather than chlorpromazine, was chosen as the reference D2-blocker. Another requirement for inclusion in the library was availability (for example, due to special storage conditions and legal status, opioid analgesics, ketamine, and 5-HT2A agonists have not yet been included in the library). The test set included drugs with similar mechanisms of action, leading us to hypothesize that the use of the method we are developing will allow us to identify similarities between compounds with similar molecular targets.

The training set, against which test-set pharmacological effects were classified, comprised previously published matrices of drug effects [7,16,17,18,24]: the NMDA antagonist dizocilpine (Targetmol Chemicals Inc., Wellesley Hills, MA, USA); D2/D3 dopamine antagonists haloperidol (OOO Velpharm, Russia) and sulpiride (AO Organica, Novokuznetsk, Russia); the M-anticholinergic tropicamide (Tocris Bioscience, Bristol, UK); the H1/5-HT2A receptor blocker hydroxyzine (Sigma-Aldrich Chemie GmbH, Taufkirchen, Germany); the acetylcholinesterase inhibitor galantamine (Sopharma AD, Sofia, Bulgaria); the α2-adrenergic agonist dexmedetomidine (Orion Corporation, Espoo, Finland); and GABA-mimetics aminophenylbutyric acid (phenibut) (Tocris Bioscience, Bristol, UK) and bromdihydrochlorophenylbenzodiazepine (phenazepam) (AO Novosibkhimpharm, Novosibirsk, Russia) (Figure 1C). Saline (OOO Grotex, Saint Petersburg, Russia) was administered as control (0.5 mL). The test set included promethazine (EGIS Pharmaceutical Plant, Budapest, Hungary); chlorpromazine (AO Valenta Pharm, Shchelkovo, Russia); ipidacrine (OOO Ellara, Pokrov, Russia); chloral hydrate (Acros Organics, Geel, Belgium); medetomidine (OOO Apicenna, Moscow, Russia); tiapride (AO Organika, Novokuznetsk, Russia); droperidol (Moscow Endocrine Plant, Moscow, Russia); diphenhydramine (Belkarolin, Vitebsk, Belarus); and controls: water for injection (Moscow Endocrine Plant, Moscow, Russia) and 20% DMSO (AO Tatkhimfarmpreparaty, Kazan, Russia) (0.5 mL). Additionally to our previous studies [11,16,17], in the present work we collected and analyzed novel recordings (Figure 1B) included in the training or test sets for α2-adrenergic blockers atipamezole and yohimbine; GABA-mimetics 5-ethyl-5-phenyl-2,4,6(1H,3H,5H)-pyrimidinetrione and 5-ethyl-5-(1-methylbutyl)-2,4,6(1H,3H,5H)-pyrimidinetrione (both Sigma-Aldrich Chemie GmbH, Germany); the adenosine receptor blocker caffeine (Sigma-Aldrich); the dopamine antagonist raclopride (Targetmol Chemicals Inc., USA); and 5-HT2 receptor blockers ritanserin and glemancerin (Targetmol Chemicals Inc., USA). In total, 78 new ECoG recordings were added to this work.

For each drug, 6–10 recordings (in random order) were obtained from different animals. Sample size was decided based on our previous studies [7,16,17,18,24]. A new drug was introduced no earlier than 2–3 days after the previous recording to avoid residual effects. Because of their long half-lives, subsequent experiments after recording 5-ethyl-5-phenyl-2,4,6(1H,3H,5H)-pyrimidinetrione and 5-ethyl-5-(1-methylbutyl)-2,4,6(1H,3H,5H)-pyrimidinetrione were delayed at least 5 days. Because of chloral hydrate’s high toxicity [26,27], animals were euthanized immediately after a 30-min ECoG recording. Notably, chloral hydrate was not used as an anesthetic but only as an experimental pharmacological agent in 10 rats during ECoG recording. The number of tests per rat (typically ≤5–6) depended on head-plug integrity (electrode connector) and the animal’s overall condition. If signs of infection were observed (pus around the head plug, porphyrin around the nose or eyes), Bicillin-3 (5000 U/kg, subcutaneously; OAO Sintez, Russia) was re-administered and the next test postponed for at least one week. Animals not improving were removed from the study. Another exclusion criterion was deterioration of background ECoG quality, typically occurring after prolonged use (>1 month). Signal deterioration typically manifested as a marked amplitude decrease across all leads or as bursts of epileptiform activity (spikes and sharp waves). In total, the analysis included data from 157 rats.

### 2.6. ECoG Signal Analysis, Dimensionality Reduction and Classification

The recordings were analyzed using Neuron-Spectr.NET omega 1.6.10.8 (OOO Neurosoft, Russia). A step-by-step explanation of the entire data processing pipeline is presented in Supplementary Materials S1. A total of 132 ECoG amplitude–spectral characteristics were calculated for six leads (FP1, FP2, C3, C4, O1, O2), including mean and maximum signal amplitudes, the standard deviation of signal amplitude, and the Lempel—Ziv compression ratio. The latter characterizes signal repeatability (i.e., higher repeatability yields a higher compression ratio). This measure is included in many biomedical-signal analysis packages and has been used to analyze rat EEG [28]. The ECoG signal was converted from the time domain to the frequency domain using the fast Fourier transform (FFT). The δ (0.5–4.0 Hz), θ (4.0–8.0 Hz), and α (8.0–14.0 Hz) frequency band, and β frequency band split into low-frequency (LF, 14.0–20.0 Hz) and high-frequency (HF, 20.0–35.0 Hz), were extracted. Mean rhythm (frequency band) amplitudes, rhythm indices, and mean powers were computed. The rhythm index was defined as the ratio of the area under the spectrum within a rhythm’s frequency range to the total area under the spectrum across the analyzed bandwidth. Average power was computed as the mean spectral power within the given frequency range. Power calculation parameters were as follows: sampling frequency 500 Hz; analysis epoch length 5 s; maximum epoch overlap 30%; and FFT points 2048. The Neuron-Spectr.NET software has developed an algorithm for automatically calculating the epoch length for the fast Fourier transform, depending on the specified epoch length for the recording-style analysis. For example, for a specified analysis epoch length of 5 s and a quantization frequency of 500 Hz, the analysis epoch length will be 2500 samples. The epoch length for the fast Fourier transform will be 2048 (two to the eleventh power) samples. Therefore, for spectral analysis of the entire epoch of 2500 samples, two fragments of 2048 samples will be analyzed, overlapping each other. The analysis results of the two fragments are averaged. In addition, mean coherence power for δ, θ, α, and β frequency bands was calculated for these lead pairs: FP1–C3, C3–O1, FP2–C4, C4–O2, FP1–FP2, FP1–C4, FP2–C3, C3–C4, C4–O1, C3–O2, O1–O2, FP1–O1, FP1–O2, FP2–O2, and FP2–O1. Coherence was estimated using the squared modulus of the cross-spectrum using a Hann window. The coherence values were calculated using this formula: Coh_xy^2(f) = |S_xy(f)|^2/(S_xx(f) · S_yy(f)), where Coh_xy^2(f)—the square of the coherence modulus for a pair of leads (x, y) at frequency f; S_xy(f)—the cross-spectrum value for the same leads at frequency f; S_xx(f) and S_yy(f)—autospectrum values (power spectral density) for leads x and y, respectively, at frequency f. Detrending and artifact rejection were performed automatically using the manufacturer’s built-in algorithms.

Amplitude–spectral results were expressed as ratios of post-administration to pre-administration values, while coherence results were expressed as post–pre differences.

Data processing and analysis were performed using the XLSTAT 2016.02.28451 add-in for MS Excel. The following datasets were used: (1) changes in ECoG amplitude–spectral characteristics; (2) changes in mean coherence powers; and (3) a combined amplitude–spectral and coherence dataset. Dimensionality reduction was performed using principal component analysis (PCA) applied to standardized data: X_norm = [X − mean(X)]/std(X)). The correlation matrix X_norm^T∙X was then decomposed. A naive Bayes classifier (NBC) was trained, and classification was performed using the derived principal components (PC1, PC2, PC3, etc.). The training set comprised effect matrices for dizocilpine, dexmedetomidine, galantamine, haloperidol, hydroxyzine, sulpiride, tropicamide, phenazepam, aminophenylbutyric acid, atipamezole, caffeine, 5-ethyl-5-phenyl-2,4,6(1H,3H,5H)-pyrimidinetrione, and NaCl.

### 2.7. Statistical Analysis

Statistical analyses were performed using XLSTAT 2016.02.28451 for MS Excel (Addinsoft, Paris, France) and GraphPad Prism 8.0.2 (GraphPad Software, San Diego, CA, USA). Researchers were blinded to the group allocation at all stages of the experiment and data analysis. Group differences shown in confusion matrices (Supplementary Materials S4) were tested for significance using the Cochran’s Q test with multiple pairwise comparisons using the McNemar (Bonferroni) procedure.

## 3. Results

### 3.1. Analysis of Changes in ECoG Amplitude–Spectral Characteristics in Rats After Administration of Training and Test-Set Drugs

Training- and test-set drugs, administered at varying doses, produced specific changes in rat ECoG amplitude–spectral characteristics. Figure 1B shows heat maps of median mean power for δ, θ, α, and β frequency bands in frontal (FP1, FP2), parietal (C3, C4), and occipital (O1, O2) leads after administration of atipamezole (ATI), caffeine (CAF), glemanserin (GMS), 5-ethyl-5-(1-methylbutyl)-2,4,6(1H,3H,5H)-pyrimidinetrione (PTB), raclopride (RPD), ritanserin (RTS), 5-ethyl-5-phenyl-2,4,6(1H,3H,5H)-pyrimidinetrione (PhBT) and yohimbine (YOH). Heat maps of changes in spectral power after administration of other drugs in the training and test samples are presented in our previously published work [11]. Each drug produced a distinct ECoG pattern, indicated by increases or decreases in mean rhythm (frequency band) power at specific leads. Dose increases did not always produce linear changes in amplitude–spectral parameters. Detailed descriptions of drug-induced changes in mean power are provided in previous studies [11,16,17,18]. Caffeine, an adenosine receptor antagonist not previously included in our library, predominantly increased mean θ and α power and had weak effects on δ and β ranges. Atipamezole (α2-adrenergic blocker) at 1 mg/kg increased θ and α power in leads C3, C4, O1, and O2. At 5 mg/kg, atipamezole increased power across all frequency bands except δ in C3 and C4; the θ power increase in parietal and occipital leads remained most pronounced. The GABAA receptor allosteric modulator 5-ethyl-5-phenyl-2,4,6(1H,3H,5H)-pyrimidinetrione at 60 mg/kg predominantly increased θ, α, and β power at FP1 and FP2, and increased α and low-frequency β (β-LF) power at C3, C4, O1, and O2. At 100 mg/kg, similar changes were observed, with an additional increase in δ-band activity in frontal leads.

### 3.2. Analysis of ECoG Coherence Changes in Rats After Administration of Training-Set Drugs

**Figure 2:**
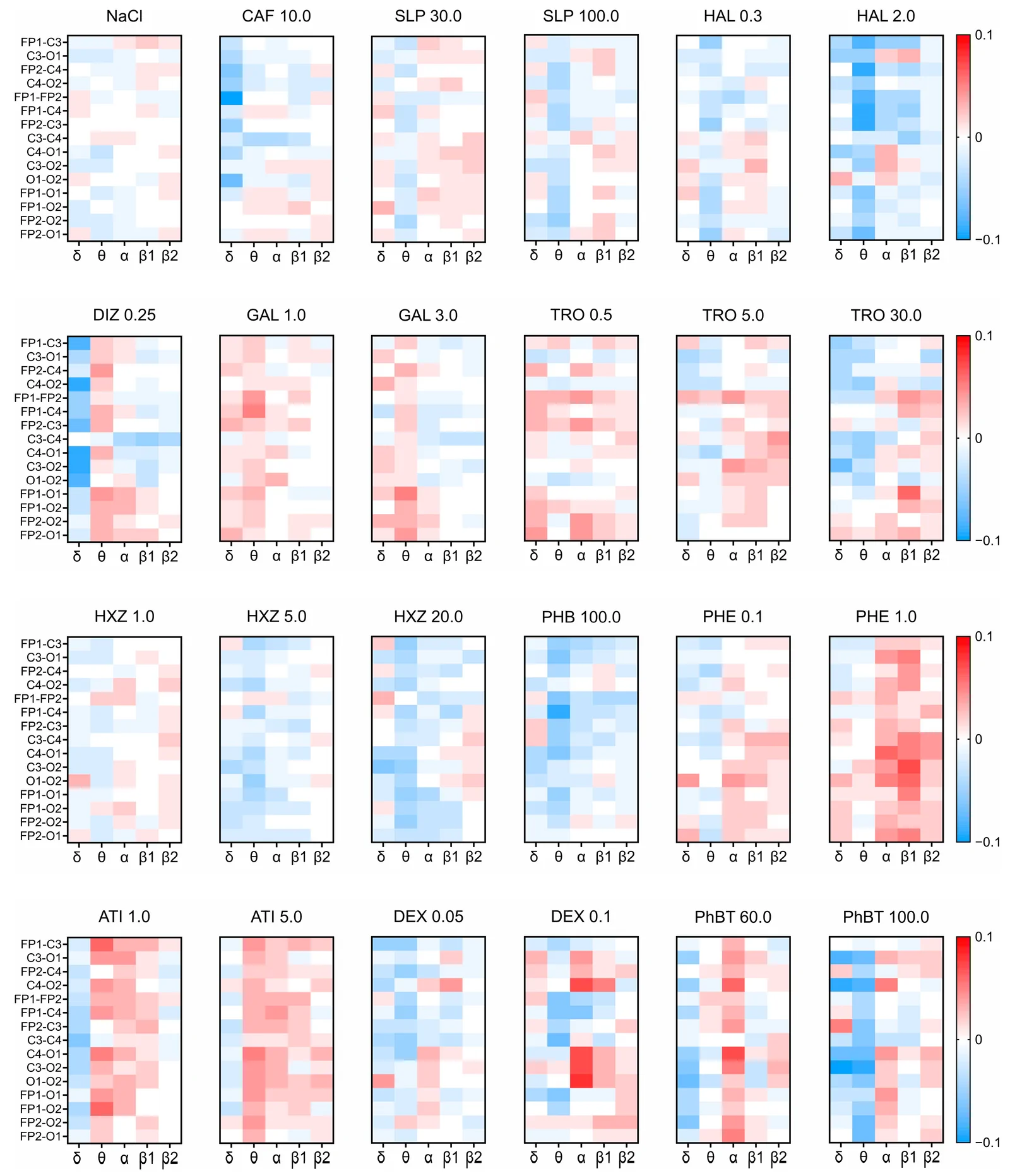
Heat maps of median changes in coherence for δ, θ, α, and β frequency bands across lead pairs after administration of saline (NaCl), caffeine (CAF), sulpiride (SLP), haloperidol (HAL), dizocilpine (DIZ), galantamine (GAL), tropicamide (TRO), hydroxyzine (HXZ), aminophenylbutyric acid (PHB), phenazepam (PHE), atipamezole (ATI), dexmedetomidine (DEX), and 5-ethyl-5-phenyl-2,4,6(1H,3H,5H)-pyrimidinetrione (PhBT). Initial data were differences between mean frequency band coherence ~20 min after administration and values before administration (~30 min of baseline recording). For each group, 6–10 recordings were made.

Administration of training-set drugs produced specific changes in coherence between electrode pairs across frequency bands (Figure 2).

For example, caffeine (10 mg/kg) reduced interhemispheric δ coherence, primarily in frontal and occipital regions. Sulpiride produced a dose-dependent decrease in θ coherence across all lead pairs. Haloperidol produced a similar but more pronounced pattern. Dizocilpine produced bidirectional effects: decreased δ coherence and increased θ coherence, without spatial localization. Galantamine predominantly increased θ coherence across lead pairs. Tropicamide at 0.5 mg/kg significantly increased mean coherence across several bands; at 5 and 30 mg/kg it decreased δ and θ synchronization across recorded regions. Hydroxyzine produced dose-dependent reductions in coherence across frequency ranges, with strongest effects in the θ band. Aminophenylbutyric acid produced effects similar to high-dose hydroxyzine. Phenazepam increased α and θ coherence across most lead pairs. Low-dose atipamezole reduced δ coherence and increased θ and α coherence; higher doses produced smaller δ changes but larger increases in θ coupling. Dexmedetomidine produced mixed effects across δ, θ, α, and β bands without a clear pattern; at the high dose, it significantly increased α synchronization between parietal and occipital regions. For 5-ethyl-5-phenyl-2,4,6(1H,3H,5H)-pyrimidinetrione, low doses decreased δ coherence and increased α coherence; at 60 mg/kg, the pattern shifted, producing decreased θ coherence across most lead pairs.

### 3.3. Data Dimensionality Reduction Using Principal Component Analysis (PCA)

PCA on amplitude–spectral characteristics yielded five principal components (PC1–PC5) that accounted for 83.05% of the variance (Supplementary Materials S2). PC1 explained 49.60% of the variance and was driven by all indicators except frequency band indices. PC2 (17.08% variance) was primarily contributed to by standard deviation across leads; amplitudes of δ, θ, β-LF, and β-HF frequency bands; indices of δ, α, β-LF, and β-HF frequency bands; and mean powers across the full band and all frequency bands except α. PC3 (8.83%) was driven by amplitudes and indices of δ and θ frequency bands across leads. PC4 (4.48%) was mainly associated with the α index in occipital leads and mean α power in frontal leads. PC5 (3.07%) was primarily formed by α indices and mean α powers across leads.

**Figure 3:**
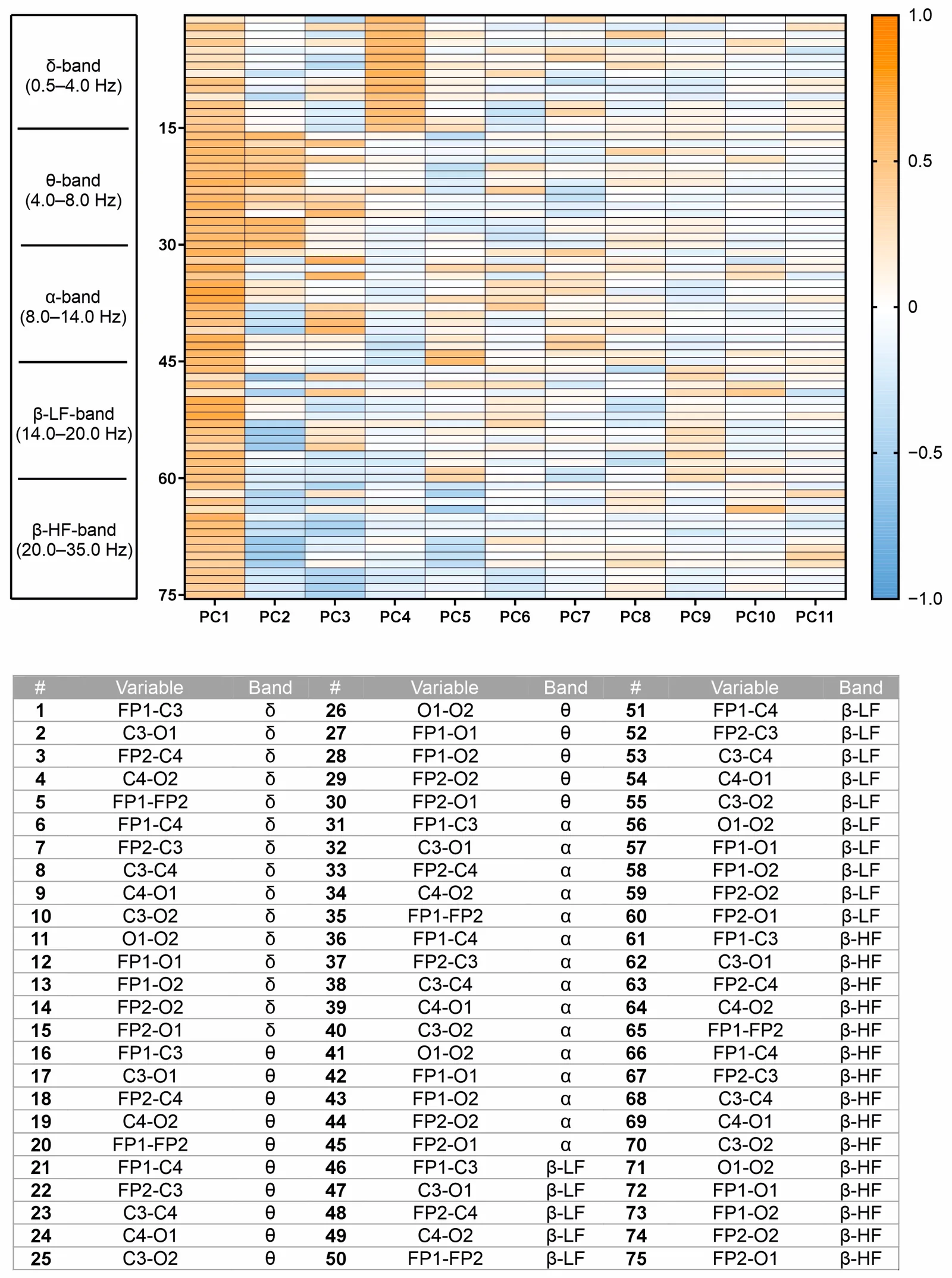
Factor loadings showing the contribution of each of the 75 ECoG coherence parameters to PCs (PC1–PC11) used in NBC analyses.

PCA on the ECoG coherence parameter array yielded 11 principal components (PC1–PC11) that explained 71.64% of the variance (Figure 3). PC1 explained 24.94% of the variance and included most parameters, except coherences for most lead pairs in the δ range and coherences between C3–O1 and C4–O2 in the β-LF and β-HF ranges. PC2 (10.90%) was driven largely by θ coherences across all lead pairs except the occipital pair (O1–O2). PC3 (8.54%) was represented by coherences between occipital and parietal electrode pairs for α and β-LF frequency bands. PC4 (7.62%) was mainly contributed to by δ-band coherences across lead pairs. PC5 (4.40%) was predominantly formed by α and β-LF coherences for FP2–O1 and FP2–O2. PC6 (3.36%) primarily reflected α and β-LF coherences between rostral and parietal electrode pairs. PC7 (3.13%) was primarily influenced by δ and α coherence between FP1 and other leads. PC8 (2.61%) was largely influenced by δ and θ coherence between FP2–C4. PC9 (2.41%) was dominated by β-LF coherence across lead pairs. PC10 (1.97%) was predominantly formed by β-LF and β-HF coherence between C4–O2. PC11 (1.71%) was primarily formed by β-HF coherences between parietal and occipital lead pairs.

**Figure 4:**
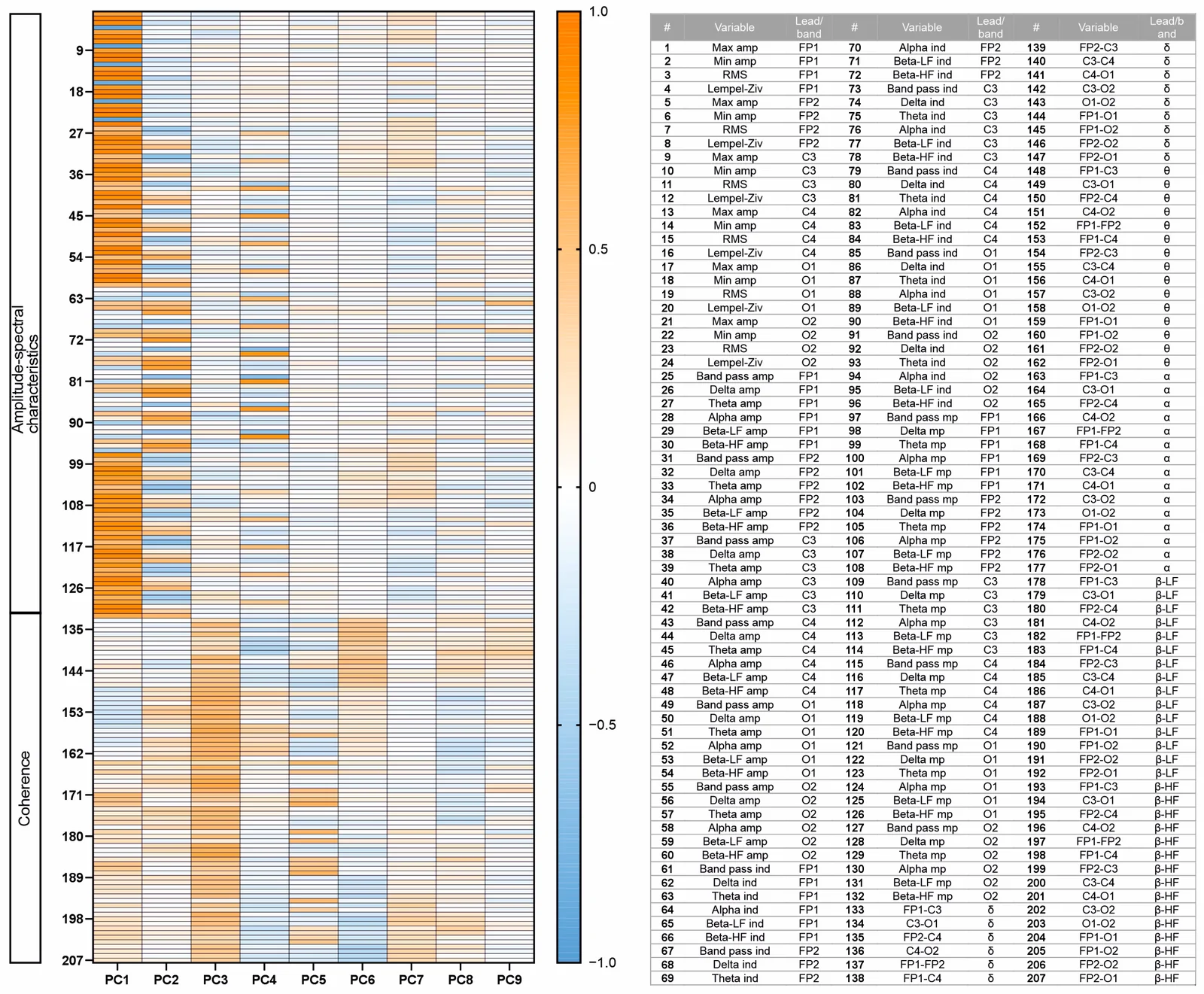
Factor loadings showing the contribution of each of the 207 amplitude–spectral and coherence parameters to PCs (PC1–PC9) used in NBC analyses.

Next, we combined two data arrays: amplitude–spectral characteristics and coherence across six ECoG leads for the full frequency spectrum. PCA on the combined array yielded nine principal components (PC1–PC9) that accounted for 73.67% of the variance (Figure 4). PC1 (32.6% variance) was driven by amplitude–spectral characteristics excluding frequency band indices. PC2 (11.30% variance) was mainly contributed to by standard deviation; amplitudes of δ, θ, β-LF, and β-HF; indices of all frequency bands except θ and the full-band index; and mean powers of all frequency bands except α across leads. PC3 (9.61%) included most coherences, except β-LF and β-HF coherences for C3–O1 and C4–O2 and most δ-range coherences. PC4 (6.67%) was largely formed by amplitudes and indices of δ and θ frequency bands and mean θ power across leads. PC5 (3.37%) was mainly influenced by α and β-LF coherence indices between parietal and occipital lead pairs. PC6 (3.03%) was primarily driven by δ-range coherences across electrode pairs. PC7 (2.72%) included mostly β-HF coherences across lead pairs except C3–C4, occipital α indices, and mean δ, θ, and α powers in frontal leads. PC8 (2.54%) was primarily formed by C3–C4 coherences in δ, β-LF, and β-HF ranges, and frontal θ indices. PC9 (1.84%) was mainly contributed to by α indices in frontal and parietal leads and δ coherence between parietal electrodes.

**Figure 5:**
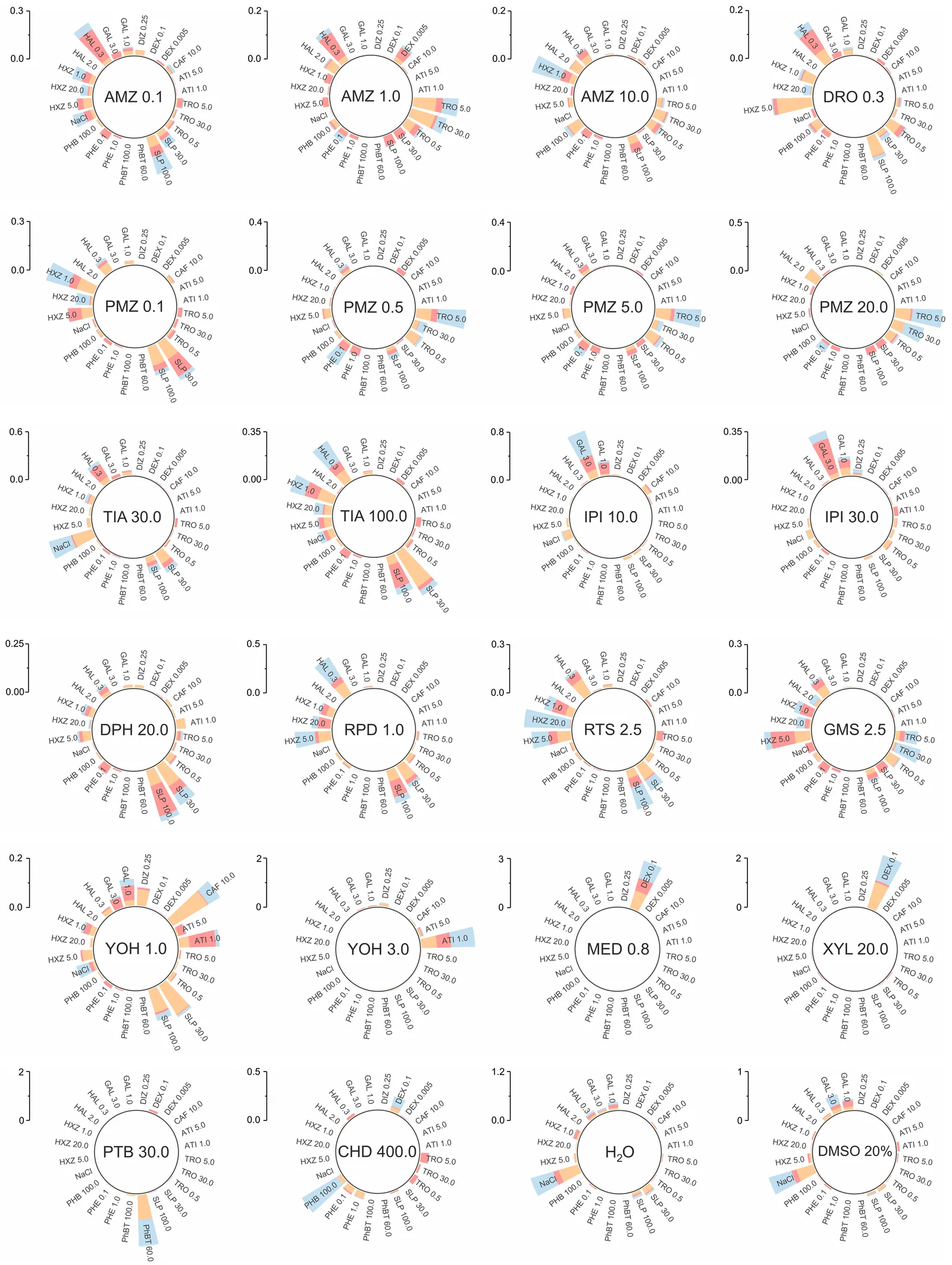
Radial diagrams show median similarity probabilities (MSP) between test-set and training-set drugs computed by the NBC. Amplitude–spectral ECoG results are orange, coherence results are red, and combined-data results are blue. Abbreviations: AMZ—chlorpromazine; DRO—droperidol; PMZ—promethazine; TIA—tiapride; IPI—ipidacrine; DPH—diphenhydramine; RPD—raclopride; RTS—ritanserin; GMS—glemancerin; YOH—yohimbine; MED—medetomidine; XYL—xylazine; PTB—5-ethyl-5-(1-methylbutyl)-2,4,6-(1H,3H,5H)-pyrimidinetrione; CHD—chloral hydrate.

The pharmacological activity of test agents was then classified using a GaussianNBC based on PC values (Supplementary Materials S3) of amplitude–spectral, coherence, or combined datasets. For each recording, probabilities were obtained for matching ECoG effects to the training set agents: saline, caffeine, sulpiride, haloperidol, dizocilpine, galantamine, tropicamide, aminophenylbutyric acid, hydroxyzine, phenazepam, atipamezole, dexmedetomidine, and 5-ethyl-5-phenyl-2,4,6(1H,3H,5H)-pyrimidinetrione. The median similarity probability (MSP) to each reference group was then calculated for test groups to infer the agents’ pharmacological profiles (Figure 5).

### 3.4. Classification of Test-Sample ECoG Effects Using Three Approaches

Chlorpromazine (AMZ) at 0.1 mg/kg resembled sulpiride 100 mg/kg (MSP = 0.06) and hydroxyzine 5 mg/kg (MSP = 0.05) based on amplitude–spectral changes. Coherence changes classified this dose as haloperidol 0.3 mg/kg (0.19); combining amplitude–spectral and coherence data classified it as sulpiride 100 mg/kg (0.10). At 1 mg/kg, amplitude–spectral and combined data indicated an anticholinergic (tropicamide-like) effect (MSP = 0.90 and 0.60, respectively). Coherence data classified this dose as similar to haloperidol 0.3 mg/kg (MSP = 0.10). At 10 mg/kg, no dominant match emerged from amplitude–spectral or coherence data alone, but the combined approach showed similarity to hydroxyzine 1.0 mg/kg (MSP = 0.11).

Droperidol (DRO) at 0.3 mg/kg was most similar by amplitude–spectral changes to hydroxyzine 5 mg/kg (MSP = 0.13) and sulpiride 100 mg/kg (MSP = 0.80). Based on coherence and the combined dataset, droperidol matched haloperidol 0.3 mg/kg (MSP = 0.90 and 0.30, respectively).

Promethazine (PMZ) at 0.1 mg/kg resembled sulpiride at 30 and 100 mg/kg (MSP = 0.11 each) and hydroxyzine 1 mg/kg (MSP = 0.10) based on amplitude–spectral changes. Coherence changes matched sulpiride 30 mg/kg (MSP = 0.12); the combined dataset matched hydroxyzine 1.0 mg/kg (MSP = 0.13). At 0.5 mg/kg, promethazine shifted toward tropicamide 5.0 mg/kg: MSP = 0.09 (amplitude–spectral), 0.05 (coherence), and 0.21 (combined). At 5.0 and 20 mg/kg, amplitude–spectral and combined approaches classified promethazine as tropicamide-like; coherence changes classified these doses as phenazepam-like and sulpiride-like, respectively.

Tiapride (TIA) at 30 mg/kg resembled saline based on amplitude–spectral and combined data (MSP = 0.23 and 0.26, respectively). Coherence data matched haloperidol 0.3 mg/kg (MSP = 0.17). At 100 mg/kg, tiapride produced stronger ECoG effects and was classified by all three approaches as sulpiride- or haloperidol-like with varying MSPs.

Ipidacrine (IPI) at both doses was classified as galantamine-like by all approaches, with highest MSPs from coherence data (MSP = 0.22 and 0.24 for IPI 10.0 and 30.0, respectively). Diphenhydramine (DPH) 20 mg/kg predominantly matched sulpiride at 30 and 100 mg/kg across data types.

Raclopride (RPD) was classified by amplitude–spectral changes as sulpiride 30 mg/kg (MSP = 0.16) and haloperidol 0.3 mg/kg (MSP = 0.13). Coherence data matched sulpiride 100 mg/kg (MSP = 0.15); combined data matched haloperidol 0.3 mg/kg (MSP = 0.22).

Amplitude–spectral changes did not yield selective matches for the 5-HT2 receptor blockers ritanserin and glemanserin; glemanserin showed a slight hydroxyzine-like predominance (MSP = 0.09). Coherence changes showed stronger similarity: ritanserin matched hydroxyzine 1 mg/kg (MSP = 0.80) and glemanserin matched hydroxyzine 5 mg/kg (MSP = 0.13). Using the combined approach, ritanserin matched hydroxyzine 20 mg/kg (MSP = 0.26); glemanserin was classified as tropicamide-like.

At 1 mg/kg, the α2-adrenergic blocker yohimbine (YOH) matched caffeine (MSP = 0.15) and sulpiride 30 mg/kg (MSP = 0.13) most closely on amplitude–spectral parameters. Coherence changes classified yohimbine 1 mg/kg as similar to atipamezole 1.0 mg/kg (MSP = 0.10). The combined data classified yohimbine 1 mg/kg as caffeine (MSP = 0.05). At 3 mg/kg, all three approaches classified yohimbine as atipamezole 1.0 mg/kg (MSP = 0.57 for amplitude–spectral, 0.52 for coherence, and 0.88 for combined data). The classifier identified both medetomidine (MED) and xylazine (XYL) as dexmedetomidine 0.1 mg/kg in all three analyses, with no notable alternatives.

The GABAA allosteric modulator PTB (5-ethyl-5-(1-methylbutyl)-2,4,6-(1H,3H,5H)-pyrimidinetrione) matched 5-ethyl-5-phenyl-2,4,6(1H,3H,5H)-pyrimidinetrione with MSP = 0.93 (amplitude–spectral) and MSP = 1.00 (combined data). Coherence-based classification identified PTB as dexmedetomidine (MSP = 0.08).

Chloral hydrate (CHD) 400 mg/kg, a GABA-mimetic general anesthetic, was classified by amplitude–spectral data as phenazepam 1.0 mg/kg and 0.5 mg/kg (MSP = 0.06 for both). Coherence data classified CHD as tropicamide 30 mg/kg (MSP = 0.07), while combined data classified it as aminophenylbutyric acid (MSP = 0.39).

Water for injection (H2O) and 20% aqueous DMSO were consistently classified as saline by all three approaches, except that coherence analysis classified 20% DMSO as similar to galantamine 1 mg/kg (MSP = 0.11).

**Figure 6:**
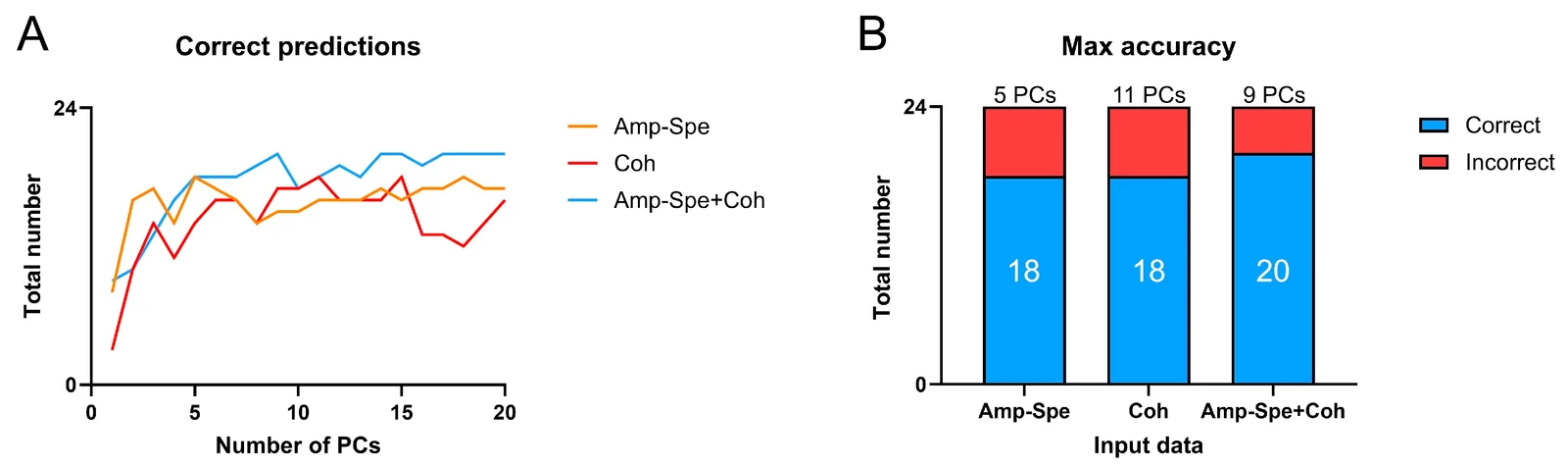
(**A**) Number of correct classifications for three approaches (amplitude–spectral, coherence, combined) across varying numbers of principal components. (**B**) Maximum classification accuracy for each approach.

To quantify classifier performance, we counted test-set groups with a “correct” similarity call. In this case for test drug the highest MSP occurred with a training-set agent sharing the same mechanism of action (see Figure 1C); MSPs with other agents were lower. For example, for water for injection, the most accurate classification was the one with saline, for ipidacrine—with galantamine, for yohimbine—with atipamezole, and so on. Comparing NBC classification accuracy across the three data types and varying numbers of principal components (PCs) revealed no statistically significant differences in the percentage of correctly classified drugs (Figure 6A). Maximum accuracy occurred at 5 PCs for amplitude–spectral data (18/24), at 11 PCs for coherence data (18/24), and at 9 PCs for the combined data array (20/24) (Figure 6B). When comparing the classification during training (confusion matrices), it was found that coherence data or adding coherence data to the data based on changes in amplitude–spectral characteristics leads to a statistically significant (*p* < 0.01 in both cases, McNemar (Bonferroni) procedure) increase in accuracy (Supplementary Materials S4).

## 4. Discussion

### 4.1. Increasing the Training Sample with Additional Pharmacological Groups Does Not Degrade the Performance of the Previously Proposed Pharmaco-EEG Classification Algorithm

In a previous study [11], amplitude–spectral ECoG changes produced correct classifications for promethazine, chlorpromazine, tiapride, ipidacrine, medetomidine, chloral hydrate, water for injection, and 20% DMSO. Importantly, adding caffeine, atipamezole, and 5-ethyl-5-phenyl-2,4,6-(1H,3H,5H)-pyrimidinetrione to the training set preserved classifier accuracy. Neither the previous nor the present amplitude–spectral data revealed the expected similarity of diphenhydramine to hydroxyzine or to tropicamide, which would have supported an H1-histamine or M-anticholinergic effect. Classification quality remained poor for droperidol, a typical antipsychotic similar to haloperidol; based on ECoG effects it was misclassified as hydroxyzine.

Raclopride, ritanserin, glemanserin, yohimbine, xylazine, and 5-ethyl-5-(1-methylbutyl)-2,4,6-(1H,3H,5H)-pyrimidinetrione—added to the test set for the first time—showed generally satisfactory results. The D2 blocker raclopride consistently resembled the antipsychotics haloperidol and sulpiride; the 5-HT2A blocker glemanserin resembled the 5-HT2A-blocking anxiolytic hydroxyzine. For the antiserotonin agent ritanserin, classification was unclear: its hydroxyzine-like similarity was offset by haloperidol- and sulpiride-like effects. Yohimbine (α2-antagonist) at 3 mg/kg matched atipamezole as expected, whereas at 1 mg/kg it matched the adenosine-receptor blocker caffeine. In this case, the similarity likely reflected comparable psychotropic effects rather than shared targets: both drugs induced stimulation, producing similar amplitude–spectral ECoG changes in rats. Therefore, this similarity should not be unambiguously interpreted as classifier failure. The sedative xylazine and the GABAA allosteric modulator 5-ethyl-5-(1-methylbutyl)-2,4,6-(1H,3H,5H)-pyrimidinetrione were clearly identified by the NBC as similar to dexmedetomidine and to 5-ethyl-5-phenyl-2,4,6-(1H,3H,5H)-pyrimidinetrione, respectively, indicating high specificity of α2- and GABAA-mediated effects on amplitude–spectral ECoG characteristics. Notably, across different component compositions, 5-ethyl-5-(1-methylbutyl)-2,4,6-(1H,3H,5H)-pyrimidinetrione resembled not only GABA-mimetic agents generally but specifically 5-ethyl-5-phenyl-2,4,6-(1H,3H,5H)-pyrimidinetrione, which targets the same GABAA subunits. These findings are consistent with prior reports indicating high specificity of GABAA receptor subunits in regulating rodent brain bioelectrical activity [29,30,31].

### 4.2. Changes in EEG/ECoG Coherence in Rodents Are Specific to Several Pharmacological Groups

Multiple research groups have used libraries of rodent EEG/ECoG recordings of pharmacological agents to classify the activity of new compounds. Notably, the team led by W. Dimpfel published initial results in the mid-1980s [31] and continued publishing through the 2010s [32,33,34,35]. In the 1990s, Krijzer et al. reported that E-10-hydroxynortriptyline (the active nortriptyline metabolite) showed predicted antidepressant and anxiolytic effects [36]. Researchers at Volgograd State Medical University used a pharmaco-EEG library of carbamazepine and saline controls to identify effects of experimental anticonvulsants [37], and an 11-agent library to recognize effects and mechanisms via cross-validation [10]. These groups used amplitude–spectral features or raw neurophysiological data as inputs to machine-learning pipelines; some transformed data into two-dimensional images for convolutional neural network input [10]. Our prior publications likewise used amplitude–spectral ECoG features [11,16,17,18,24].

Despite these important results, improved approaches to increasing machine-learning accuracy are still needed. Connectivity analysis—particularly coherence, which quantifies similarity between oscillatory activity across regions within specific frequency bands—may be valuable for identifying drug-specific EEG effects. Several studies have used coherence analysis to identify pharmacological effects on rodent brain bioelectrical activity. Páleníček et al. [19] showed that the psychedelic 2C-B (4-bromo-2,5-dimethoxyphenethylamine) produced dose- and time-dependent coherence changes in a pharmaco-EEG analysis. Primary findings were as follows: 10 mg/kg impaired connectivity, whereas 50 mg/kg increased high–frequency connectivity. The 10 mg/kg coherence decrease resembled effects reported for classic hallucinogens (psilocin, mescaline, DOB, LSD). The authors proposed that decreased theta- and gamma-range coherence may reflect impaired information processing, including disrupted perception and spatial processing. These changes likely underlie altered locomotor behavior, stereotypies, and impaired prepulse inhibition. The same study reported increased coherence after amphetamine, which likely reflects drug-induced hyperlocomotion and increased nucleus-accumbens dopamine release.

Guidera et al. [38] assessed coherence dynamics among the prefrontal cortex, parietal cortex, and central thalamus during stepwise increases in sevoflurane concentration. During induction, coherence increased in the beta/gamma-to-low–frequency range between PFC–CT and within PFC connections. Loss of movement was accompanied by a sharp decrease in beta–gamma–to–low-frequency coherence between PFC and CT. At concentrations producing loss of righting reflex, high delta-range coherence predominated across all leads.

Zhao et al. [21] reported that the NMDA antagonist dizocilpine (a schizophrenia model) decreased 55–95 Hz coherence in frontal, parietal, and occipital cortices.

These examples support that psychotropic drugs from different pharmacological classes induce significant coherence changes in rats. Published data suggest these coherence changes can be drug-specific. To test this, we compared average coherence power across drugs from different pharmacological classes and evaluated whether these data enable accurate classification with the previously proposed algorithm.

### 4.3. Using Coherence Changes Improves Classification Accuracy for Some Pharmacological Classes While Reducing It for Others

When coherence changes were used as NBC input (as with amplitude–spectral features), maximum accuracy was 18/24. Promethazine and chlorpromazine produced the expected similarities to sulpiride, haloperidol, hydroxyzine, and tropicamide. These MSPs varied by dose, likely reflecting differing degrees of dopamine, histamine, and muscarinic receptor blockade. Unlike the amplitude–spectral approach, coherence analysis showed droperidol to be strongly similar to the structurally and pharmacologically closest training drug, haloperidol. The atypical antipsychotic tiapride consistently resembled sulpiride and haloperidol. Notably, the NBC detected haloperidol-like signatures for this dataset even at low doses, indicating greater sensitivity of coherence data for haloperidol. Average ECoG coherence changes were particularly specific for acetylcholinesterase inhibitors. Both ipidacrine doses were unambiguously classified as galantamine-like, and this similarity persisted across numbers of principal components—a robustness not seen with amplitude–spectrum analysis. Coherence analysis did not detect H1-histamine or muscarinic (M) anticholinergic signatures for diphenhydramine. Raclopride, ritanserin, and glemanserin were identified as sulpiride-like or hydroxyzine-like; classification accuracy was particularly better for ritanserin. Similar to tiapride, coherence analysis showed low-dose yohimbine resembling the pharmacologically related atipamezole. Medetomidine, xylazine, water for injection, and 20% DMSO were classified as expected (dexmedetomidine or saline) and showed MSP values comparable to amplitude–spectral–based classifications. However, despite coherence-data advantages, NBC produced implausible classifications for 5-ethyl-5-(1-methylbutyl)-2,4,6-(1H,3H,5H)-pyrimidinetrione and for chloral hydrate. Across nearly all numbers of principal components, the GABA modulator was identified as dexmedetomidine and chloral hydrate as tropicamide. The GABA-modulator—dexmedetomidine similarity may reflect shared sedative effects, but the chloral-hydrate–tropicamide similarity remains unexplained.

### 4.4. Combining Amplitude–Spectral Changes and Coherence Yields a Modest Increase in Classification Accuracy

NBC classifications based on amplitude–spectral features and on coherence showed equal overall accuracy but differing sensitivities to the same drugs. We combined the two datasets and compared this combined approach with the two individual approaches. The combined approach maintained classification accuracy for chlorpromazine and promethazine, revealing dose-dependent similarities to sulpiride, tropicamide, and hydroxyzine. At promethazine doses ≥ 0.5 mg/kg, a predominantly tropicamide-like effect was observed. Consequently, the 20 mg/kg promethazine similarity to the cataleptogenic haloperidol dose (2.0 mg/kg) seen with amplitude–spectral data was no longer detectable in the combined dataset. Droperidol showed the expected similarity to haloperidol. However, the MSP equaled that for hydroxyzine, outperforming the coherence-only classification. Classification of both tiapride doses matched the amplitude–spectral–based results. The combined dataset preserved NBC sensitivity to AChE inhibitors: both ipidacrine doses were classified as galantamine-like.

As with the other approaches, no similarity was detected between diphenhydramine and tropicamide or hydroxyzine. Diphenhydramine may nonetheless exhibit dopamine-blocking activity, suggested here by three instances of similarity to sulpiride. We found no direct literature on diphenhydramine as a dopamine receptor blocker; however, Tanda et al. reported that diphenhydramine blocks the DAT transporter and increases nucleus-accumbens dopamine in rats [39]. Given sulpiride’s presynaptic dopamine-receptor blockade and resultant synaptic dopamine increase [40], the sulpiride–diphenhydramine similarity may reflect both drugs’ net stimulation of dopaminergic neurotransmission. This hypothesis is speculative and requires experimental confirmation.

Raclopride produced satisfactory results, showing similarity to haloperidol and to hydroxyzine. Compared with the other approaches, the combined array provided superior classification for these agents. For the 5-HT2A blocker glemanserin, the combined approach produced an inconsistent tropicamide-like classification.

Classification of α2 agonists and antagonists from the combined dataset closely matched amplitude–spectral–based classifications and was generally successful.

For the GABA mimetic and chloral hydrate, and for controls (water for injection and 20% DMSO), the highest expected similarities were: GABA modulator → 5-ethyl-5-phenyl-pyrimidinetrione; chloral hydrate → aminophenylbutyric acid; controls → water for injection. Combining datasets corrected the coherence-only misclassification of chloral hydrate as tropicamide.

### 4.5. Limitations of the Study and Future Prospects

No statistically significant differences were found among the three classification methods applied to different ECoG datasets in rats. This may reflect the relatively small number of drug classes included. For example, the library currently excludes opioid receptor agonists/antagonists, cannabinoids, several serotonin-receptor–active agents, and other classes. Expanding the library to include these classes would provide a more detailed assessment of NBC sensitivity across pharmacologies. Notably, training data were drawn mainly from clinically used drugs. This choice permits reliance on published effects and more deliberate dose selection for rats. However, these agents often lack selectivity for single pharmacological targets. Therefore, we plan to replace some library entries with selective agonists/antagonists for specific neurotransmitter receptors.

Although coherence remains widely used [19,21,23,37,41,42], more recent connectivity metrics have been proposed, including phase-locking value (PLV), mutual information (MI), phase-lag index (PLI), and Granger causality [43,44]. Granger causality assesses effective connectivity, enabling inference about the directionality and nature (excitatory vs. inhibitory) of influence between cortical regions. Future work to develop an effective pharmaco-EEG screening tool using PCA and NBC should compare the sensitivity of these connectivity and coherence metrics to the actions of distinct drug classes.

It is also worth noting that increased training accuracy is not evidence of improved generalization. However, this is an indicator of progress and the adequacy of the model. An increase in training accuracy during training indicates that the model is truly learning, and not simply “memorizing” noise in the data. Among other things, this speaks in favor of the “sufficiency” of the size of our samples and the parameters used in training. It is worth noting a significant drawback of our work: our design does not let us directly compare training accuracy and the classification accuracy of the test set (we use different groups of drugs in the training and test sets). A possible solution to this problem could be to use drugs similar to those in the training set for the test set.

## 5. Conclusions

Principal component analysis combined with a naïve Bayes classifier detected ECoG effects of multiple psychotropic drug classes, including D2/D3 blockers, muscarinic (M) agents, H1-histamine blockers, 5-HT2A blockers, AChE inhibitors, GABAergic agonists, and α2-adrenergic agonists/antagonists. Using amplitude–spectral features, average coherence power, or their combination, the approach correctly classified 18–20 of 24 test groups. Although differences were not statistically significant, the naïve Bayes classifier applied to the combined dataset yielded a greater number of consistent similarities. These results support the use of the combined amplitude–spectral and coherence dataset as a more promising approach for future pharmaco-EEG screening of novel psychotropic compounds.

## Data Availability

The data supporting the findings of this study are available in Supplementary Files.

## Supplementary Materials

The following supporting information can be downloaded at: https://www.mdpi.com/article/10.3390/mps9050123/s1, Below is the link to the electronic supplementary material. S1. S2. S3. S4.
